# A Lightweight Dangerous Liquid Detection Method Based on Depthwise Separable Convolution for X-Ray Security Inspection

**DOI:** 10.1155/2022/5371350

**Published:** 2022-01-18

**Authors:** Dongming Liu, Jianchang Liu, Peixin Yuan, Feng Yu

**Affiliations:** ^1^College of Information Science and Engineering, Northeastern University, Shenyang 110819, China; ^2^State Key Laboratory of Synthetical Automation for Process Industries, Northeastern University, Shenyang 110819, China; ^3^School of Mechanical Engineering and Automation, Northeastern University, Shenyang 110819, China

## Abstract

For personal safety and crime prevention, some research studies based on deep learning have achieved success in the object detection of X-ray security inspection. However, the research on dangerous liquid detection is still scarce, and most research studies are focused on the detection of some prohibited and common items. In this paper, a lightweight dangerous liquid detection method based on the Depthwise Separable convolution for X-ray security inspection is proposed. Firstly, a dataset of seven common dangerous liquids with multiple postures in two detection environments is established. Secondly, we propose a novel detection framework using the dual-energy X-ray data instead of pseudocolor images as the objects to be detected, which improves the detection accuracy and realizes the parallel operation of detection and imaging. Thirdly, in order to ensure the detection accuracy and reduce the computational consumption and the number of parameters, based on the Depthwise Separable convolution and the Squeeze-and-Excitation block, a lightweight object location network and a lightweight dangerous liquid classification network are designed as the backbone networks of our method to achieve the location and classification of the dangerous liquids, respectively. Finally, a semiautomatic labeling method is proposed to improve the efficiency of data labeling. Compared with the existing methods, the experimental results demonstrate that our method has better performance and wider applicability.

## 1. Introduction

At present, nondestructive testing technology has been widely applied in various fields [1–4], among which the application of X-ray detection technology in airports, customs, railway stations, and other transportation departments reduces criminal behavior effectively. However, the technology requires security inspectors to determine whether prohibited items are hidden in baggage. During the rush hours, the passing frequency of baggage increases greatly, and the security inspectors have to complete the detection in a very short time. Moreover, the images of prohibited items are often distorted or corrupted. These factors make detection more difficult and bring great challenges to security detection. Up to now, manual detection has been widely used in the field of X-ray security detection, but this method mainly relies on the experience of security inspectors. Meanwhile, the detection results of different security inspectors are also different. The accuracy of manual detection cannot be assured [5]. Accordingly, a fast and effective automatic object detection method for X-ray security inspection is significant.

In the early stage of the automatic object detection method for X-ray security inspection, some feature extraction algorithms are often used. Common feature extraction algorithms include Scale-invariant Feature Transform (SIFT), Histogram of Oriented Gradient (HOG), Haar-like features (Haar), etc. [6]. In addition, for the single-energy X-ray image, a method using the visual vocabulary and an occurrence structure generated from a training dataset were proposed in [7]. A new approach called Adaptive Sparse Representation (XASR+) was proposed in [8], and several patches were extracted from X-ray images to construct representative dictionaries in this method. For the dual-energy X-ray pseudocolor images, Franzel et al. [9] used the visual vocabulary and the SVM classifier to detect handguns in hand luggage. Wang [10] et al. proposed a method by combining the Taruma feature based on the contourlet transform and the histogram, which applied the random forests classifier to classify these features from the illegal objects. In [11], Uroukov et al. used textural signatures to recognize and characterize materials. However, due to the wide variety of objects in X-ray security inspection, the fast detection requirement, the noise, the perspective imaging, the geometric distortion, and the objects placed closely together, these detection methods based on these manual features are not satisfactory.

In recent years, deep learning in image analysis and processing, especially the convolutional neural networks (CNNs), has achieved great success [12–14]. Compared with the manual feature extraction algorithms, the methods based on deep learning could automatically provide the most descriptive and differentiated features for each classification by training on a large dataset for a long time. For the object detection of natural optical images based on the convolutional neural network, the methods are mainly divided into two classifications. One is the two-stage method, such as R-CNN [15], Fast R-CNN [16], Faster RCNN [17], R-FCN [18], and FPN [19]. Such methods generate a set of candidate region suggestions firstly, and these candidates are classified, filtrated, and refined again. So far, the accuracy of the two-stage method still has been the highest among the object detection methods. The other is the one-stage method that directly predicts the classification and bounding box by a single convolutional network, such as YOLO methods [20–23] and SSD [24]. This kind of method has a faster detection speed, but the accuracy is lower than the two-stage method. Both of them have achieved brilliant achievements in the object detection of natural optical images and have been applied in various fields [25–27].

Compared with natural optical images, object detection in X-ray security inspection is still a huge challenge since X-ray images are very different from natural optical images. In [28], Mery et al. used transfer learning to classify three kinds of threat objects based on X-ray images and compared the experimental results with traditional computer vision methods. The experimental results showed that the X-ray image classification method based on deep learning is effective and potential. Akcay et al. [29] tested and evaluated several existing networks and object detection methods for six classifications of objects based on X-ray images, and the result showed the object detection methods with deep learning are better than the methods without deep learning. In [30], the researchers proposed a method that is more accurate and robust when dealing with the dense cluttered background in X-ray security inspection. The method adopted a specific data enhancement technique, the feature enhancement modules, and the multiscale fusion regions of interest (ROI). However, the current researches focused on a few common types of prohibited items due to the difficulty of establishing and extending a complete dataset. In response to this question, Zhang et al. [31] proposed a method of X-ray prohibited items image generation using Generative Adversarial Networks (GANs). In [32], Zhu et al. proposed a method based on Cycle GAN to transform the item natural images into X-ray images. These methods provide a new research direction for dataset expansion and lay a foundation for more accurate object detection in X-ray security inspection. Meanwhile, the high hardware requirements of these methods based on deep learning also limit their application.

At present, most researches are focused on the detection of some prohibited and common items, such as guns, knives, batteries, laptops, and bottles. The shapes and materials of these items are diverse. The above researches have made some contributions to the object detection of these items. Due to the high similarity of liquid pseudocolor images, the above researches can only detect bottles, not liquid types. However, for some special security occasions, only detecting the bottle is not enough. In these occasions, it is necessary to detect whether the liquid is harmful or even detect the type of the liquid. As far as we know, there is no research using dual-energy X-ray data to detect dangerous liquids. A possible solution to the classification of dangerous liquids is Energy dispersive X-ray diffraction (EDXRD). In [33], Zhong et al. found that Energy dispersive X-ray scattering profile is unique to each specific liquid material through experiments with three types of liquids. Tianyi et al. utilized EDXRD with hybrid discriminant analysis to classify the liquids in [34]. However, there are many problems in these researches. For instance, the sample must be a small dose, the container has specific requirements, the detection time is long, and the sample must be placed in a fixed position.

To solve these problems, we design an effective, lightweight dangerous liquid detection method, and it does not require high hardware requirements. The main contributions are as follows:

- We propose a novel framework using the dual-energy X-ray data instead of pseudocolor images as the objects to be detected, which improves the detection accuracy and realizes the parallel operation of detection and imaging
- We design a lightweight object location network as the backbone network of object location, which ensures the object location accuracy and has fewer parameters and less computational consumption
- We design a lightweight dangerous liquid classification network as the backbone network of classification, which has higher accuracy, fewer parameters, and less computational consumption
- A dataset of seven common dangerous liquids containing multiple postures in two detection environments is established
- A semiautomatic labeling method is proposed to reduce the cost of manual labeling

The rest of the paper is organized as follows: Section 2 describes the creation and processing of the dataset. In Section 3, the proposed method is described.  Section 4 presents the experiments and results. Finally, Section 5 concludes the paper and discusses some directions for future work.

## 2. Dataset

**Figure 1:**
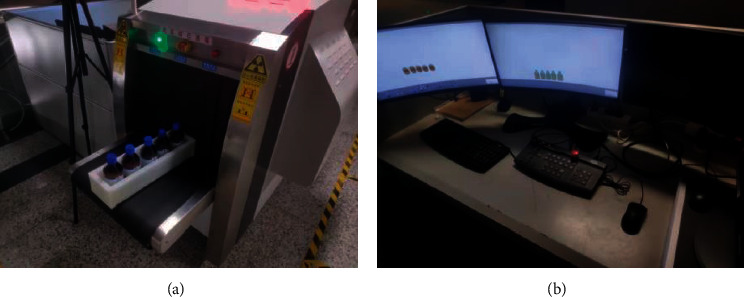
(a) The X-ray security inspection equipment. (b) The control station.

The dual-energy X-ray method has been widely used in X-ray security inspection systems [35]. In the method, the high-energy and low-energy data are converted into a pseudocolor image by a lookup table to facilitate the interpretation of the detected objects. In order for security inspectors to better distinguish the material of the detected object, orange represents organic matter, green represents mixture, and blue represents inorganic matter in the pseudo color image. In this paper, the dual-energy X-ray data used in this work are manually collected using X-ray security inspection equipment from Shenyang DT Inspection Equipment Co. Ltd. in China. The X-ray tube voltage and current are 140 kV and 0.75 mA, respectively. The X-ray security inspection equipment is shown in Figure 1. The value range of the dual-energy X-ray data is 0-15200. The size of each data is 600 × 600 × 2.

**Figure 2:**
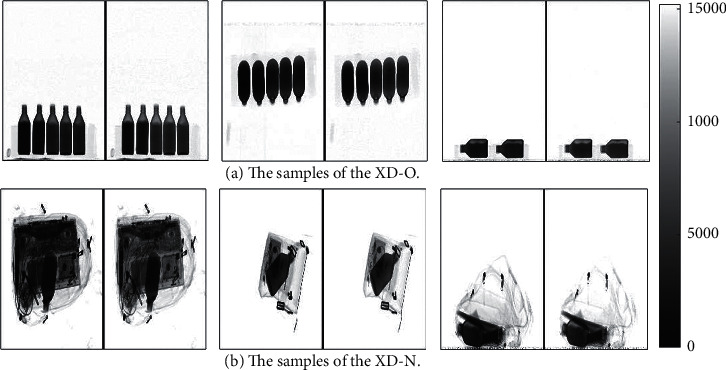
Some grayscale images of the dual-energy X-ray data. (The left side of each image is the low-energy image and the right side is the high-energy image.) (a) The sample of the XD-O. (b) The sample of the XD-N.

**Figure 3:**
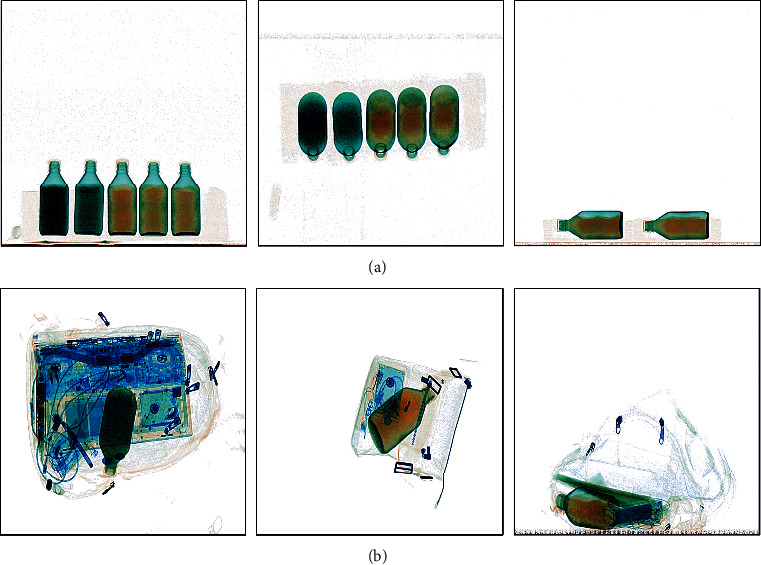
Some pseudocolor images of the dual-energy X-ray data. (a) The sample of the XD-O. (b) The sample of the XD-N.

**Figure 4:**
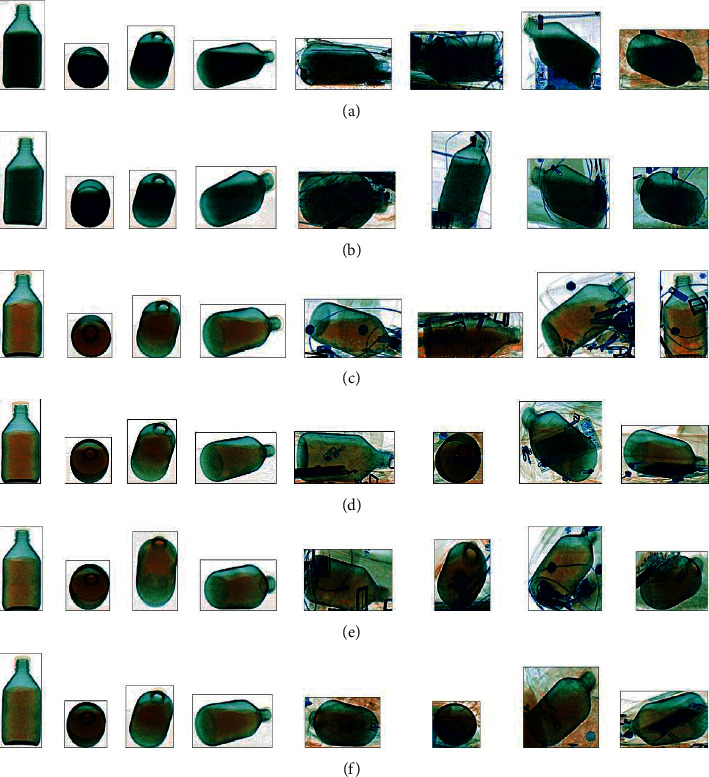
Some pseudocolor images of the different liquids. (a) Some samples of H2SO4. (b) Some samples of HCL. (c) Some samples of C7H8. (d) Some samples of CH3OH. (e) Some samples of CH3COCH3. (f) Some samples of C2H5OH.

A total of 7 kinds of dangerous liquids samples purchased from Sinopharm Chemical Reagents Shenyang Co., Ltd. without further purification are measured for our work. The samples are as follows: ethanol (≥75%), ethanol (≥95%, CP), methanol (≥99.7%, GR), acetone (99.5%, AR), methylbenzene (≥99.5%, AR), sulfuric acid (95–98%), and hydrochloric acid (36–38%). For descriptive convenience, the names of the liquids are substituted by chemical formulas in the latter part of this paper. Our X-ray dataset is divided into two parts to simulate two detection environments. One is to simulate the open-bag security inspection termed XD-O. This type of inspection is common in important situations such as airports. The other is to simulate the normal security inspection termed XD-N. For the XD-O, the samples with different postures are placed in the foam box and transferred to the security inspection machine through the conveyor belt. A total of 2318 dual-energy X-ray data are collected in the XD-O. For the XD-N, to simulate the real situation, the different baggage with the samples is packed and then sent into the security inspection machine. A total of 3596 dual-energy X-ray data are collected in the XD-N. The grayscale images of the dual-energy X-ray data are shown in Figure 2. The pseudo color images observed by the security inspectors are shown in Figure 3.  Figure 4 shows the samples of the different liquids in the foam boxes and the real baggage.

From Figure 4, we can find that the similarity between sulfuric acid and hydrochloric acid is high and the other four liquids are also very similar under the conditions of our imaging method. Moreover, the pseudocolor images of these liquids may be more similar under the different levels of obscuration of different items. Considering this situation, it is difficult for the conventional algorithms based on the pseudocolor images to achieve the detection of these liquids. Therefore, our method uses the dual-energy X-ray data containing more detailed information instead of the pseudo color images as the objects to be detected. As a crucial part of the training network process, the dataset will directly affect the performance of deep learning methods. In order to improve the accuracy and robustness of our method, our dataset was augmented to 17308 samples by the translation, replication, and random noise injection.

## 3. Methods

In this section, the method proposed in this paper is described in detail. It is well known that some object detection methods, such as YOLO methods and Faster RCNN, are applied to various object detection tasks. However, the number of parameters and computational consumption is large for our detection task. In order to achieve a good balance between the number of parameters, computational consumption, and detection accuracy for our detection task, we propose a lightweight dangerous liquid detection method for X-ray security inspection (DLDX) with higher accuracy, fewer parameters, and less computational consumption. Firstly, we design the framework of the DLDX. Secondly, we design two lightweight networks as the backbone networks of the DLDX to achieve the object location and classification, respectively. Then, we design a semiautomatic labeling method for our dataset to improve the efficiency of data labeling. Finally, we give the training strategy of the DLDX.

### 3.1. The Framework of the DLDX

Our DLDX is designed based on the two-stage method. For the existing two-stage detection method, the candidate region suggestions are generated by the specific pooling operation (such as Roipooling and Roialign) on the candidate areas of the feature map, and then these candidates are classified, filtrated, and refined again. Although this approach is successful in the object detection of natural images, it causes a large amount of information loss in the process of object extraction, which is unfavorable to the dangerous liquid detection. At the same time, a large number of candidates also bring a large amount of computational consumption, which limits the application of this method.

**Figure 5:**
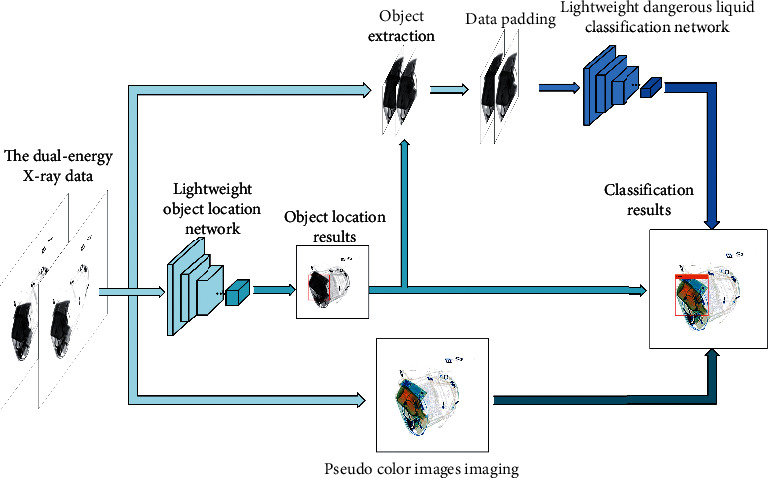
The framework of the DLDX.

To solve these problems, we propose the DLDX. The dual-energy X-ray data are used instead of pseudocolor images as the objects to be detected in the DLDX, which improves the detection accuracy and realizes the parallel operation of detection and imaging. The detection process is mainly divided into three parts: object localization, object extraction, and classification. Firstly, an object location network is used to precisely locate dangerous liquids. Secondly, to ensure the integrity of the extracted objects information, the objects are directly extracted from the dual-energy X-ray data through the position information of the objects output by the lightweight object location network, instead of extracting the objects on the feature map like the Roipooling and the Roialign. Then, in order to improve the classification accuracy, the extracted objects are padded to a fixed size. Considering the distance between the objects, the size of the objects, and the rationality of the network design, we use 15200 to pad the extracted objects to 256 × 192 instead of directly extracting the data with the size of 256 × 192 from the dual-energy X-ray data. Thirdly, these padded data are classified through a dangerous liquid classification network. Eventually, a pseudocolor image with the detection result is presented to the security inspectors. The overall architecture of the proposed method is shown in Figure 5.

### 3.2. A Lightweight Object Location Network

The design of the backbone network is significant for the accurate prediction of object locations. Therefore, this subsection focuses on developing a lightweight object location network of dangerous liquids that has both high detection accuracy and low computational cost. For the two-stage detection methods, taking Faster RCNN as an example, the Region Proposal Network (RPN) generates the candidate region suggestions with various scales and ratio aspects on the feature map to coarsely regress the bounding box location and classify the foreground and background. And then these candidates are fed into the next network to refine the bounding box location and classification accuracy. Finally, the bounding box coordinates, class labels, and classification accuracy are output. Although this method ensures detection accuracy, it also brings a large amount of calculation consumption due to the processing of a large number of candidates from the RPN. It is worth noting that the image data is three-dimensional with a value range of 0–255, while our data is two-dimensional with a value range of 0–15200. This means that our dataset is quite different from the ImageNet dataset and the fine-tuning of the networks pretrained on the ImageNet dataset is impossible. Meanwhile, designing a new network with lots of parameters makes training difficult and requires a huge dataset. Based on the above discussion, we design a lightweight object location network fitting our dual-energy X-ray dataset. It has fewer parameters and can be trained from scratch with our dataset.

**Figure 6:**
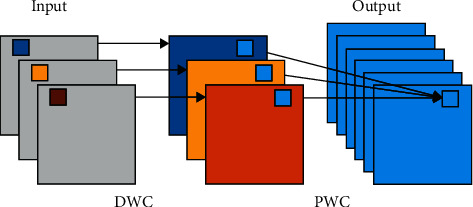
The convolution process of the Depthwise Separable convolution.

In our lightweight object location network, the Depthwise Separable convolution composed of the Depthwise convolution (DWC) and the Pointwise convolution (PWC) is employed. The DWC is operated by channel-wise fashion and the PWC is the standard convolution with 1 × 1 kernels. The operation process of the Depthwise Separable convolution is shown in Figure 6. To illustrate the advantages of the Depthwise Separable convolution, we compare the Depthwise Separable convolution and the standard convolution in terms of the number of parameters (Params) and the number of multiply-accumulate operations (Madds).

Given an input feature map *X*_in_ ∈ *R*^*H*×*W*×*C*^ and an output feature map $X_{\text{out}}\in R^{\tilde{H}\times \tilde{W}\times \tilde{C}}$, the ratio of Madds between the Depthwise Separable convolution and the standard convolution can be represented as follows:

(1) $$\begin{array}{c} R_{\text{Madds}}=\frac{{\text{Madds}}_{ds}}{{\text{Madds}}_{s}}=\frac{D_{k}\times D_{k}\times C\times \tilde{H}\times \tilde{W}+C\times \tilde{C}\times \tilde{H}\times \tilde{W}}{D_{k}\times D_{k}\times C\times \tilde{C}\times \tilde{H}\times \tilde{W}}\\ =\frac{1}{\tilde{C}}+\frac{1}{D_{k}\times D_{k}}, \end{array}$$

where  *D*_*k*_ is the size of the convolution kernel, Madds_*ds*_ is the Madds of the Depthwise Separable convolution, and Madds_*s*_ is the Madds of the standard convolution. The ratio of Madds between the Depthwise Separable convolution and the standard convolution can be represented as follows:

(2) $$\begin{array}{c} R_{\text{Params}}=\frac{{\text{Params}}_{ds}}{{\text{Params}}_{s}}=\frac{D_{k}\times D_{k}\times C+C\times \tilde{C}}{D_{k}\times D_{k}\times C\times \tilde{C}}\\ =\frac{1}{\tilde{C}}+\frac{1}{D_{k}\times D_{k}}, \end{array}$$

where Params_*ds*_ is the Params of the Depthwise Separable convolution and Params_*s*_ is the Params of the standard convolution.

**Figure 7:**
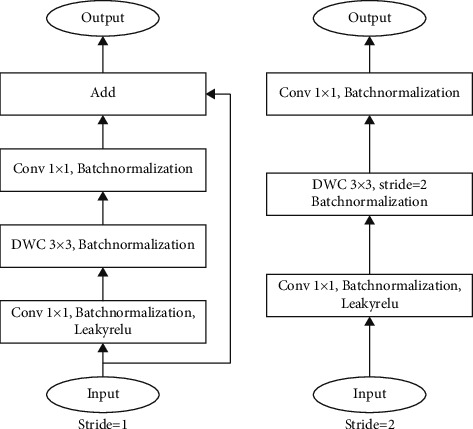
The structure of the improved inverted residual block.

We can find the Params and Madds of the Depthwise Separable convolution have been greatly reduced. Based on the computational advantage of the Depthwise Separable convolution, it is employed in some successful lightweight networks, such as MobilenetV1 [36] and MobilenetV2 [37]. In MobilenetV2, the inverted residual block was proposed based on the DWC and the residual network. As the Depthwise convolution is operated by channel-wise fashion, the feature information can only be transferred in one channel. Meanwhile, the ReLu6 activation function causes a large amount of information loss in the inverted residual block. Therefore, we improve the inverted residual block in MobilenetV2, as shown in Figure 7. In the improved inverted residual (IIRS) block, we replace the ReLu6 activation function of the first PWC with the LeakyRelu and remove the activation functions after the DWC, which ensures the effective transmission of the information.

Our lightweight object location network is designed based on the IIRS block. The architecture of the lightweight object location network is shown in Table 1. First, a 3 × 3 Conv + BatchNormalization + LeakyRelu block is used to reduce the dimension of the input data and extract roughly the features. As the network needs to be trained from scratch, the use of the Batchnormalization and the LeakyRelu makes the training of the network easier. Then, four IIRS blocks are applied to extract the object features accurately with fewer parameters. Subsequently, in order to reduce the computational consumption, only two 3 × 3 Conv + BatchNormalization + LeakyRelu blocks are used to extract the features more accurately.

Next, we use the K-means clustering algorithm to obtain the size of the anchor boxes. A total of six anchor boxes are obtained for getting more precise object positions. It is well known that feature maps with the larger size contain richer location information. In order to make the prediction of the object location more accurate and take into account the computational consumption, the size of the feature map is set as 75 × 75. That is to say, the input data is divided into 75 × 75 grids. In each grid, the prediction information from the last convolutional layer consists of the position offset  (*t*_*x*_, *t*_*y*_), width offset *t*_*w*_, height offset *t*_*h*_ and confidence score *p* of each anchor box. Using the sigmoid and exp function, we can obtain the final output (*t*_*fx*_, *t*_*fy*_, *t*_*fw*_, *t*_*fh*_, *p*_*f*_)=(*S*(*t*_*x*_), *S*(*t*_*x*_), *e*^*t*_*w*_^, *e*^*t*_*h*_^, *S*(*p*)), where *S*(·) is the sigmoid function and *e* is the natural logarithm. The actual coordinates (*x*, *y*, *w*, *h*) and the normalized confidence score *P* can be obtained through the following equation:

(3) $$\begin{array}{c} \left[\begin{array}{c} x\\ y\\ w\\ h\\ P \end{array}\right]=\left[\begin{array}{c} \left(t_{fx}+c_{x}\right)\times F\\ \left(t_{fy}+c_{y}\right)\times F\\ A_{w}\times t_{fw}\\ A_{h}\times t_{fh}\\ p_{f} \end{array}\right], \end{array}$$

where *F* is the minification factor of the feature map, *F*=600/75 (600 is the input size and 75 is the feature map size) in this paper, (*c*_*x*_, *c*_*y*_) is the coordinate in the upper left corner of each grid in the feature map, (*A*_*w*_, *A*_*h*_) is the width and height of the anchors.

At the end of the lightweight object location network, multiple overlapping candidate boxes will be suggested, and we must choose the best one from these candidate boxes for each object. Therefore, Soft-NMS algorithm [38] is used to update the score of each boundary box. Define *B*={*b*_1_, *b*_2_,…, *b*_*N*_} as the set of the candidate boxes and *V*={*v*_1_, *v*_2_,…, *v*_*N*_} as the corresponding set of the scores. The choice criterion in Soft-NMS can be written as follows:

(4) $$\begin{array}{c} V_{i}=\left\{\begin{array}{cc} V_{i}, & \text{IOU}\left(b_{m},b_{i}\right)<T,\\ V_{i}\left(1-\text{IOU}\left(b_{m},b_{i}\right)\right), & \text{IOU}\left(b_{m},b_{i}\right)\geq T, \end{array}\right. \end{array}$$

where *b*_*m*_ is the candidate box which has the highest score, *b*_*i*_ is the initial detection candidate box, and *T* is the threshold value of the intersection over union (IOU). IOU is the ratio of the intersection and union of two candidate boxes. When the IOU values of the candidate boxes are smaller than the threshold *T*, the scores of the candidate boxes remain unchanged. Soft-NMS assigns lower scores to the neighboring candidate boxes, whose IOU values are bigger than the threshold *T* until the final prediction boxes are selected instead of removing them.

The loss function of the lightweight object location network *L*_object_ consists of the coordinate error *L*_boxes_ and the confidence score error *L*_score_. The coordinate error can be defined as follows:

(5) $$\begin{array}{c} L_{\text{boxes}}=\lambda_{\text{cood}}\overset{M}{\underset{i=1}{\sum }}\overset{N}{\underset{j=1}{\sum }}I_{ij}^{obj}\left[{\left(t_{fxij}-{\hat{t}}_{fxij}\right)}^{2}+{\left(t_{fyij}-{\hat{t}}_{fyij}\right)}^{2}+{\left(t_{fwij}-{\hat{t}}_{fwij}\right)}^{2}+{\left(t_{fhij}-{\hat{t}}_{f}hij\right)}^{2}\right], \end{array}$$

the confidence score error is defined as follows:

(6) $$\begin{array}{c} L_{\text{score}}=-\overset{M}{\underset{i=1}{\sum }}\overset{N}{\underset{j=1}{\sum }}I_{ij}^{obj}\lambda_{obj}{\left(p_{fi}-{\hat{p}}_{fi}\right)}^{2}+\lambda_{\text{noobj}}\overset{M^{2}}{\underset{i=1}{\sum }}\overset{N}{\underset{j=1}{\sum }}I_{\text{IOU}<\text{Thresh}}{\left(p_{fi}\right)}^{2}, \end{array}$$

and the final loss function can be expressed as follows:

(7) $$\begin{array}{c} L_{\text{object}}=L_{\text{boxes}}+L_{\text{score}}, \end{array}$$

where *λ*_cood_ is the weight coefficient of the coordinate error (set as 1), *λ*_obj_ is the weight coefficient of the confidence error for the grids with an object (set as 5), *λ*_noobj_ is the weight coefficient of the confidence error for the grids with the IOU less than the threshold (set as 0.5), *M* is the number of the grids (set as 75 × 75), *N* is the number of the anchor boxes in each gird (set as 6), $\left({\hat{t}}_{fxij},{\hat{t}}_{fyij},{\hat{t}}_{fwij},{\hat{t}}_{fhij},{\hat{p}}_{fij}\right)$ is the true value for (*t*_*fxij*_, *t*_*fyij*_, *t*_*fwij*_, *t*_*fhij*_, *p*_*fij*_), *I*_*ij*_^obj^ is 1 if the *j* th anchor box predicted by grid *i* is responsible for the prediction (0 otherwise), and *I*_MaxIOU<Thresh_ is 1 if the IOU of the *j* th anchor box predicted by grid *i* is less than the threshold (0 otherwise).

### 3.3. A Lightweight Dangerous Liquid Classification Network

After the object location and the data padding, these padded data are classified through a dangerous liquid classification network in our DLDX. The design of the classification network is very significant and it directly affects the performance of our DLDX. At present, some public CNN networks can be used for the image feature extraction, such as Darknet [20, 21], Resnet [14], MobilenetV2 [37], MobilenetV3 [39], and SqueezeNet [40]. These networks have outstanding performance in the object detection of natural images, and they are usually used as the backbone networks for feature extraction when dealing with practical problems in the engineering field. Meanwhile, transfer learning is often adopted to solve these problems. However, transfer learning is not applicable for our dataset because our dataset is completely different from the ImageNet dataset. In order to ensure the accuracy of the classification and reduce the computational consumption and the number of parameters, we designed our lightweight dangerous liquid classification network based on the IIRS block and the Squeeze-and-Excitation (SE) block [41].

**Figure 8:**
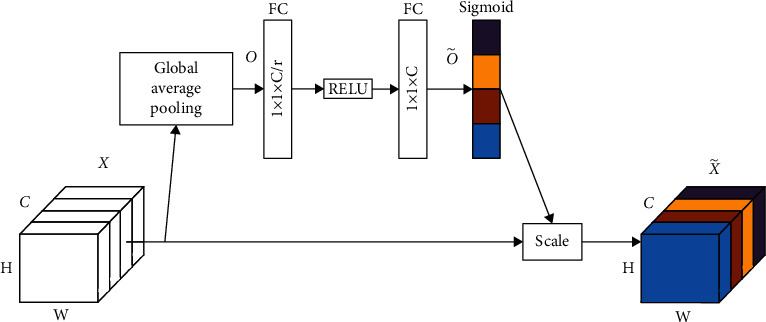
The structure of the Squeeze-and-Excitation block.

The SE block can be understood as feature maps recalibrated according to channels. This recalibration makes the network ignore those channels with less meaningful information and focus on the ones that provide more meaningful information. The structure of the SE block is shown in Figure 8. Given an input feature map *X* ∈ *R*^*H*×*W*×*C*^, we can get the recalibrated feature map $\tilde{X}\in R^{H\times W\times C}$ through the SE block. *X* and $\tilde{X}$ can be expressed as *X*=[*x*_1_, *x*_2_,…, *x*_*C*_] and $\tilde{X}=\left[{\tilde{x}}_{1},{\tilde{x}}_{2},\ldots ,{\tilde{x}}_{C}\right]$. Firstly, the global average pooling is used to generate a 1 × 1 × *C* feature map *O*=[*o*_1_, *o*_2_,…, *o*_*C*_] to express *X* in general. This process can be expressed as follows:

(8) $$\begin{array}{c} O_{k}=\frac{1}{H\times W}\overset{H}{\underset{i=1}{\sum }}\overset{W}{\underset{j=1}{\sum }}x_{k}\left(i,j\right),\;k=1,2,\ldots ,C. \end{array}$$

Secondly, the channel-wise dependencies $\tilde{O}=\left[{\tilde{o}}_{1},{\tilde{o}}_{2},\ldots ,{\tilde{o}}_{C}\right]$ are extracted using fully connected (FC) layers and nonlinearity layers. The connection mode is shown in Figure 8. We can obtain the following:

(9) $$\begin{array}{c} \tilde{O}=S\left(W_{2}\sigma \left(W_{1}O\right)\right), \end{array}$$

where *σ* represents the Relu activation function, **W**_1_ ∈ *R*^(*C*/*r*)×*C*^ and **W**_2_ ∈ *R*^*C*×(*C*/*r*)^ are the weights of the fully connected layers, *r* is a ratio parameter. Finally, the recalibrated feature map $\tilde{X}$ can be obtained by the following equation:

(10) $$\begin{array}{c} \tilde{X}=\text{Scale}\left(\tilde{O},X\right)=\left[{\tilde{o}}_{1}x_{1},{\tilde{o}}_{2}x_{2},\ldots ,{\tilde{o}}_{C}x_{C}\right], \end{array}$$

where Scale(·) refers to channel-wise multiplication between the scalar $\tilde{O}$ and the feature map *X*.

**Figure 9:**
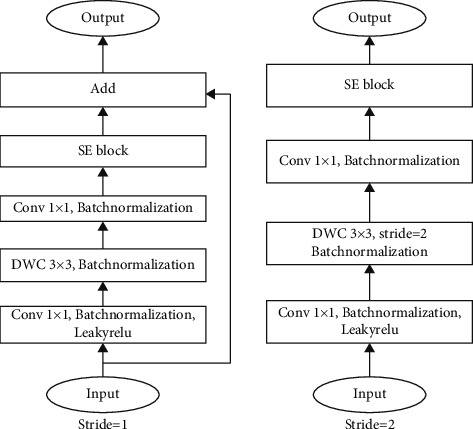
The structure of the IIRS + SE block.

Following, we combine the IIRS block and the SE block into an IIRS + SE block. The SE block is placed behind the last PWC in the IIRS + SE block. This approach can recalibrate the information of the IIRS block better. The structure of the IIRS + SE block is shown in Figure 9.  Table 2 shows the architecture of our lightweight dangerous liquid classification network.

First, like the lightweight object location network, a 3 × 3 Conv + BatchNormalization + LeakyRelu block is used to reduce the dimension of the input data and extract the features roughly. Second, on the premise of effectively extracting features, in order to reduce the computational consumption and the difficulty of network training, the IIRS + SE blocks with stride = 1 and stride = 2 are combined, as shown in Table 2. After the last IIRS + SE block, a 1 × 1 convolution filter is adopted to increase the dimension and enrich the information of the extracted feature. And then, the average global pooling is adopted to reduce computational consumption and prevent overfitting, like most networks. Finally, full connection and softmax are used to output the final classification results. We use the cross entropy function as the loss function:

(11) $$\begin{array}{c} L_{\text{class}}=-\overset{K}{\underset{k=1}{\sum }}{\hat{P}}_{ck}\log\left(P_{ck}\right), \end{array}$$

where *K* is the number of classes, ${\hat{P}}_{ck}$ is the actual label of the input data, and *P*_*ck*_ is the probability that the Softmax layer predicts the input data belonging to the class *k*.

### 3.4. A Semiautomatic Labeling Method

Information labeling is the basis of building deep learning models and a necessary process for supervised machine learning algorithms. For the public datasets, the most common labeling method is manually labeled by the crowdsourcing business model. However, the datasets in the security field are highly professional and confidential and cannot be transmitted via the Internet. This leads to a significant increase in the cost of manual labeling. In order to reduce the cost of manual labeling of our dangerous liquid dataset, a semiautomatic labeling method based on active learning is designed to improve the efficiency of dataset labeling, and the lightweight object location network is fine-tuned in the process.

To introduce our algorithm clearly, the augmented dual-energy X-ray dataset is defined as *U*={*U*_1_, *U*_2_}, where *U*_1_ is the labeled dataset, $U_{2}=\left\{{\tilde{U}}_{1},{\tilde{U}}_{2},\ldots ,{\tilde{U}}_{n}\right\}$ is the dataset with classification labels but without location labels, and it is divided into *n* datasets to be labeled. The semiautomatic labeling algorithm is shown in Algorithm 1.

In our semiautomatic labeling method, the initial state of the dual-energy X-ray dataset with labels $\bar{U}$ is *U*_1_. Next, the initialized object location network is trained on the labeled dataset *U*_1_, and the trained object location network is used to predict the subset in *U*_2_. For each subset of *U*_2_, the samples with low confidence and the undetected samples are selected and put into the manually labeled dataset *U*_*m*_, and the samples with high confidence are updated with the labels of the prediction. In the end, the manually labeled dataset and updated dataset are combined into $\bar{U}$ for fine-tuning the object location network until all subsets of *U*_2_ are processed.

### 3.5. Training Strategy of the DLDX

For our DLDX, two networks need to be trained. The object localization network can be trained in the semiautomatic labeling process and can also be directly trained by using the labeled dataset. After training the object localization network, the trained object localization network is used to extract the dangerous liquid objects from the dual-energy X-ray dataset as the training dataset of the dangerous liquid classification network. In the extraction process, the object location network outputs the bounding boxes after shielding the Soft-NMS algorithm. Then the IOU values of the generated bounding boxes and corresponding labeled boxes are calculated and sorted, and up to 10 bounding boxes are selected for each object. These extracted objects are padded to 256 × 192 to form the training dataset of the dangerous liquid classification network. Finally, the dangerous liquid classification network can be trained on the dataset.

## 4. Experimental Results and Analyses

In this section, we first test our semiautomatic labeling method, then compare our DLDX with the existing methods and analyze the experimental results. The experiments are run on a GPU system with the following specifications: Intel Core i9-10900k CPU, 64 GB RAM and NVIDIA GeForce GTX 3090 GPU.

### 4.1. Evaluation Criteria

In this paper, average precision (AP) and mean average precision (*mAP*) are used to evaluate the performance of the methods. In addition, *m*IOU is the average of the IOU values of all predicted boxes and object boxes, and it is also used to evaluate the methods. Precision and Recall are calculated using the following equations:

(12) $$\begin{array}{c} \mathrm{Pr}=\frac{\text{TP}}{\text{TP}+\text{FP}}, \end{array}$$

(13) Re=TPTP+FN,

where TP is the number of true positive samples, FP is the number of false-positive samples, and FN is the number of false-negative samples. High precision indicates high accuracy of detection results, and high Recall means fewer missed objects in the detection process. Average precision can be calculated as follows:

(14) AP=111∑Re∈0,0.1,0.2,……,1maxRe˜:Re˜≥RePrRe˜,

where Pr(Re) is the measured precision at recall Re. Subsequently, *mAP* can be defined as follows:

(15) $$\begin{array}{c} mAP=\frac{1}{K}\overset{K}{\underset{k=1}{\sum }}AP_{k}. \end{array}$$

### 4.2. Results and Discussion

In order to verify the effectiveness of our semiautomatic labeling method, we manually labeled our dataset and randomly selected some data to form the unlabeled dataset. Then, we used our semiautomatic labeling method to train our lightweight object location network and label the unlabeled dual-energy X-ray dataset. In the process, *U*_1_ contained 8440 samples, *U*_2_ was divided into four subsets (each subset contained 2000 examples), and the confidence score threshold *T* was set as 0.9. Adaptive moment estimation (Adam) optimization algorithm was used for the training of all networks and the batch size was 8. For *U*_1_, the initial learning rate was 0.001, the exponential decay rate for the first moment estimate was 0.9, the exponential decay rate for the second moment estimate was 0.999, and the max epoch was 100. For the process of fine-tuning, the learning rate was 0.0001, the max epoch was 20, and other parameters were the same as above. Meanwhile, we also trained our lightweight object location network on the manually labeled dataset for comparison with our method. The results are given in Table 3. The results show the *mIOU* of the trained lightweight object location network with our semiautomatic labeling is only 0.011 lower than the network trained on manually labeled dataset. The small gap is entirely acceptable. Therefore, our semiautomatic labeling method is effective.

And then, we prioritized training our DLDX on the XD-O. The samples in the XD-O all have simple backgrounds and the training results can better represent the feature extraction and classification abilities of our DLDX for the different liquids. In the process, Adam optimization algorithm was used. The batch size was 64, the max epoch was 30, the initial learning rate was 0.001, and other parameters were the same as above. Moreover, to further evaluate the performance of our method, we also trained the existing object detection methods and the existing lightweight CNN networks as the backbone networks of the DLDX to compare with our DLDX. Considering the existing methods were designed based on images, we converted the XD-O into a corresponding pseudocolor images dataset and trained the existing methods on the XD-O and the pseudocolor images dataset. The detection results of each method are shown in Table 4.

Meanwhile, in order to compare the complexity of each method, the Params and Madds of each method are shown in Table 5. As the Params and Madds of Faster RCNN are much more than others, they are not given in Table 5. In addition, the Params and Madds of the method based on the dual-energy X-ray dataset and pseudocolor images dataset are almost equal. Therefore, only the Params and Madds of the methods based on the dual-energy X-ray data are given in Table 5.

From Table 4, we can find that the *mAP* of the methods using the dual-energy X-ray data as the input is generally better than the methods using the pseudocolor images as input. This proves that it is reliable for using the dual-energy X-ray data as the objects to be detected. In terms of the structures of the methods, using the same backbone network, the *mAP* and the *mIOU* of our DLDX are higher than those of YOLOV4_tiny and Faster RCNN. It is worth noting that compared with using YOLOV4_tiny, the Params of using the DLDX are reduced by 45% for mobileNetV2 and 20% for mobileNetV3. Since our DLDX classifies objects after locating them, the Madds of the DLDX is related to the number of objects. For the DLDX with mobileNetV2 and mobileNetV3, their Madds are the same as using YOLOV4_tiny when each sample contains ten objects, the Madds are reduced by 50% when each sample contains three objects and the Madds are reduced by 67% when each sample contains one object. In terms of the backbone networks, the *mAP* of the DLDX with mobileNetV2 and mobileNetV3 is almost equal to our method, but the Params of our method are reduced by about 80%. Compared with mobileNetV2 and mobileNetV3, the Madds of our lightweight dangerous liquid classification network is reduced by 74% and 83%, respectively. According to the above analysis, it can be concluded that our DLDX can accomplish highly accurate dangerous liquid detection in the open-bag security inspection and the fewer Params and Madds of our DLDX can also greatly reduce the hardware requirements, which makes our DLDX have wider applicability.

To further verify the performance of our DLDX in complex environments, we trained our DLDX on the XD-N. According to the experimental results based on the XD-O, YOLOV4_tiny_MobileNetV2_X-ray, YOLOV4_tiny_MobileNetV3_X-ray, DLDX_MobileNetV2, and DLDX_MobileNetV3 were selected to compare with our DLDX. The detection results are shown in Table 6. From Table 6, we can find that the *mAP* and the *m*IOU of these methods decrease on the XD-N with complex background. However, the *mAP* of our DLDX is still able to reach 90.96%, and using the same backbone network, the *mAP* and the *m*IOU of our DLDX is higher than those of YOLOV4_tiny. Among these methods, our DLDX still has the highest *m*IOU and *mAP*, although the AP of HCL, CH3OH, CH3COCH3, and C2H5OH are slightly lower than that of the DLDX using MobileNetV2 and MobileNetV3 as backbone networks.

**Figure 10:**
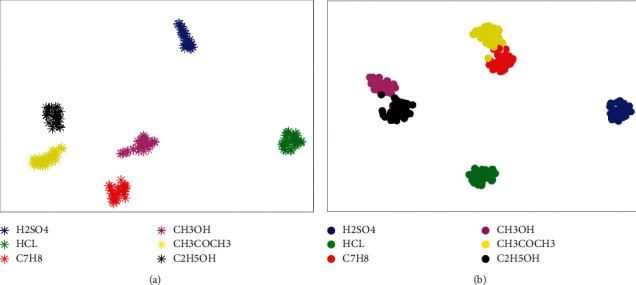
The feature visualization results based on t-SNE for our method. (a) XD-O. (b) XD-N.

Except that, we adopted the t-distributed stochastic neighbor embedding method (t-SNE) [42] as the feature visualization method to demonstrate the feature extraction ability of our method. The results are shown in Figure 10. The visualization results indicate that our method can extract better features from the samples with simple backgrounds in the open-bag security inspection to distinguish the different liquid classes. The quality of the extracted features for the samples with complex backgrounds is slightly inferior to that of the samples with simple backgrounds, which is the reason for the decrease in the accuracy of identifying the samples with complex backgrounds. According to the above analysis, it can be concluded that our method is more suitable for the detection of dangerous liquids and has wider applicability than other methods.

## 5. Conclusion

In this paper, an effective lightweight, dangerous liquid detection method for X-ray security inspection termed DLDX is proposed. The innovation is mainly reflected in three major aspects. First, a novel detection framework using the dual-energy X-ray data as the objects to be detected is proposed to improve the detection accuracy and realize the parallel operation of detection and imaging. Different from the framework of existing two-stage methods, the objects are directly extracted from the dual-energy X-ray data and padded to a fixed size as candidates in our DLDX, which ensures the integrity of the information. Second, in order to ensure the detection accuracy and reduce the computational consumption and the number of parameters, a lightweight object location network and a lightweight dangerous liquid classification network using the Depthwise Separable convolution and the SE block are designed. Third, a semiautomatic labeling method is proposed for our dataset to improve the efficiency of data labeling. To demonstrate the effectiveness of our method, we first verify the effectiveness of our semiautomatic labeling method through the experiments. And then, we conduct a series of experiments to compare our DLDX with the existing methods. The experimental results demonstrate that our proposed method has fewer Params and Madds and higher detection accuracy than the existing methods.

In future work, we will focus on expanding the types of dangerous liquids and containers and improving our method with dual-view technology to detect dangerous liquids more accurately.

## Figures and Tables

**Algorithm 1:**
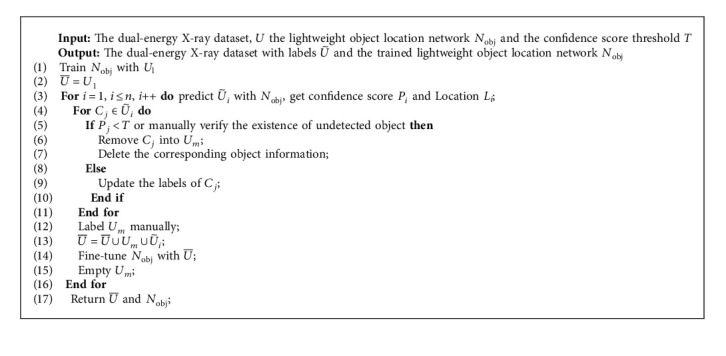
Semiautomatic labeling method.

**Table 1:** The architecture of the lightweight object location network.

Input	Operator	*C*	*T*	Stride
600 × 600 × 2	Conv + Bn + LeakyRelu	16	—	2
300 × 300 × 16	IIRS block	32	1	2
150 × 150 × 32	IIRS block	32	3	1
150 × 150 × 32	IIRS block	64	3	2
75 × 75 × 64	IIRS block	64	2	1
75 × 75 × 64	Conv + Bn + LeakyRelu	64	—	1
75 × 75 × 64	Conv + Bn + LeakyRelu	64	—	1
75 × 75 × 64	Conv	30	—	1
75 × 75 × 30	Sigmoid + Exp function	—	—	—
Output	75 × 75 × 30			

*T* is the multiplier of the input channel and *C* is the number of the last PWC channels.

**Table 2:** The architecture of the lightweight dangerous liquid classification network.

Input	Operator	*C*	*T*	Stride	r
256 × 192 × 2	Conv + Bn + LeakyRelu	16	—	2	—
128 × 96 × 16	IIRS + SE block	16	2	2	16
64 × 48 × 16	IIRS + SE block	16	3	1	16
64 × 48 × 16	IIRS + SE block	32	3	2	16
32 × 24 × 32	IIRS + SE block	32	3	1	16
32 × 24 × 32	IIRS + SE block	64	3	2	16
16 × 12 × 64	IIRS + SE block	64	3	1	16
16 × 12 × 64	IRS + SE block	128	3	2	16
8 × 6 × 128	IRS + SE block	128	4	1	16
8 × 6 × 128	Conv + Bn + LeakyRelu	512	—	1	—
8 × 6 × 512	Globalpooling	—	—	—	—
1 × 1 × 512	Fully connected	6	—	—	—
Output	Softmax				

**Table 3:** The object location results.

Method	MIOU	AP
Semiautomatic labeling	0.878	100
Manually labeled dataset	**0.889**	100

**Table 4:** The detection results of the different methods on the XD-O.

Method	MIOU	MAP (%)	AP(%)					
			H2SO4	HCL	C7H8	CH3OH	CH3COCH3	C2H5OH
YOLOV 4_tiny_X-ray	0.892	94.34	**100**	**100**	92.84	89.66	92.40	91.16
YOLOV4_tiny_MobileNetV2_X-ray	0.884	93.78	**100**	**100**	90.06	92.93	90.62	89.06
YOLOV4_tiny_MobileNetV3_X-ray	0.884	94.33	**100**	99.22	92.19	88.83	93.75	91.97
YOLOV4_tiny_image	0.892	92.68	98.44	96.88	91.28	91.41	89.80	88.24
YOLOV4_tiny_MobileNetV2_image	0.884	91.74	95.12	**100**	91.39	89.02	82.88	92.04
YOLOV4_tiny_MobileNetV3_image	0.885	92.38	93.75	98.44	92.19	86.72	89.70	93.48
FasterRCNN_MobileNetV2_X-ray	0.819	91.92	91.95	98.99	88.28	92.44	87.49	92.37
FasterRCNN_MobileNetV3_X-ray	0.821	92.03	91.35	98.44	92.19	90.59	86.72	92.88
FasterRCNN_MobileNetV2_image	0.821	89.95	92.91	98.44	86.67	88.25	85.91	87.49
FasterRCNN_MobileNetV3_image	0.822	90.73	93.75	98.44	92.13	85.12	86.64	88.27
DLDX_MobileNetV2	**0.902**	98.05	**100**	99.22	90.63	**100**	**98.44**	**100**
DLDX_MobileNetV3	**0.902**	97.43	**100**	**100**	90.24	99.82	96.88	97.66
DLDX	**0.902**	**98.28**	**100**	**100**	**96.02**	99.88	96.88	96.88

**Table 5:** The Params and Madds of the different methods.

Method	Params (m)	Madds
YOLOV4_tiny	5.9	7144 m
YOLOV4_tiny_MobileNetV2	4.3	3477 m
YOLOV4_tiny_MobileNetV3	3.4	2832 m
DLDX_MobileNetV2	2.4	856 m + 290 m × *n*
DLDX_MobileNetV3	2.8	856 m + 195 m × *n*
DLDX	0.4	856 m + 50 m × *n*

m denotes million and *n* is the number of objects.

**Table 6:** The detection results of the different methods on the XD-N.

Method	MIOU	MAP (%)	AP(%)					
			H2SO4	HCL	C7H8	CH3OH	CH3COCH3	C2H5OH
YOLOV4_tiny_MobileNetV2_X-ray	0.824	83.75	97.62	90.89	68.71	83.63	73.91	87.73
YOLOV4_tiny_MobileNetV3_X-ray	0.835	84.12	95.18	90.91	66.44	88.03	75.24	88.89
DLDX_MobileNetV2	**0.876**	89.14	97.56	**94.86**	86.64	80.84	84.47	90.44
DLDX_MobileNetV3	**0.876**	90.56	**100**	92.80	84.14	**85.60**	**89.77**	**91.05**
DLDX	**0.876**	**90.96**	**100**	94.57	**88.03**	85.59	87.28	90.27

## Data Availability

The dataset used to support the findings of this study was supplied by Shenyang DT Inspection Equipment Co., Ltd. in China, under license, and the dataset involving security cannot be shared.
